# Genomic Selection for Optimum Index with Dry Biomass Yield, Dry Mass Fraction of Fresh Material, and Plant Height in Biomass Sorghum

**DOI:** 10.3390/genes11010061

**Published:** 2020-01-05

**Authors:** Ephrem Habyarimana, Marco Lopez-Cruz, Faheem S. Baloch

**Affiliations:** 1 CREA Research Center for Cereals and Industrial Crops, via di Corticella 133, 40128 Bologna, Italy; 2 Crop, Soil, and Microbial Sciences Department, Michigan State University, 1066 Bogue St, East Lansing, MI 42824, USA; 3 Department of Field Crops, Faculty of Agricultural and Natural Sciences, Abant Izzet Baysal University, 14030 Bolu, Turkey

**Keywords:** *Sorghum bicolor*, *Sorghum halepense*, genomic selection, genomic prediction, optimum index, index selection, biomass, yield, plant height, GBS SNP

## Abstract

Sorghum is one of the world’s major crops, expresses traits for resilience to climate change, and can be used for several purposes including food and clean fuels. Multiple-trait genomic prediction and selection models were implemented using genotyping-by-sequencing single nucleotide polymorphism markers and phenotypic data information. We demonstrated for the first time the efficiency genomic selection modelling of index selection including biofuel traits such as aboveground biomass yield, plant height, and dry mass fraction of the fresh material. This work also sheds light, for the first time, on the promising potential of using the information from the populations grown from seed to predict the performance of the populations regrown from the rhizomes—even two winter seasons after the original trial was sown. Genomic selection modelling of the optimum index selection including the three traits of interest (plant height, aboveground dry biomass yield, and dry mass fraction of fresh mass material) was the most promising. Since the plant characteristics evaluated herein are routinely measured in cereal and other plant species of agricultural interest, it can be inferred that the findings can be transferred in other major crops.

## 1. Introduction

Relying on fossil fuels is a major challenge to a world struggling to adapt and mitigate climate change [1]. In this context, biomass sorghum is a cereal crop that can play an important role for sustainable and environment friendly farming, as it is particularly resilient to drought stress [2] and is more energy efficient than most plant species of agricultural interest, including maize and sugarcane [1]. Sorghum biomass can be used to produce several types of green fuels including biogas and bioethanol, with reduced greenhouse emissions, which are less polluting for the environment relative to fossil fuels [3]. Biomass yield is the primary trait in biomass sorghum, as it measures productivity and profitability of the farmer. Sorghum biomass yields are positively correlated with plant height, maturity, and the concentration of the dry mass [1]. Breeding efforts to increase biomass production should therefore mostly focus on these plant characteristics.

Efficient breeding requires also knowledge-based selection strategies that efficiently exploit available phenotypic and genotypic information that existed in the crop of interest. Since the economic value of the final product in a breeding program depends on several traits [4,5], it can be inferred that selecting the parents for the next generation based on several different plant characteristics can improve genetic gain. Genomic selection (GS) showed good results in the selection of complex quantitative traits like yields [6,7] and it has been successfully implemented in plant breeding and animal husbandry [8,9]. The main features of the GS approach is the use of algorithms that combine phenotypes and high-density marker data to predict genetic merit upon which superior unphenotyped candidates are selected [10,11]. This attribute is particularly interesting as it can reduce the costs associated with evaluating trials, shorten generation interval, and bypass the extensive field progeny testing that are otherwise required to select parental lines to be used in crossing blocks [12]. Several genomic selection methods have been developed and successfully implemented in plant breeding programs and in animal husbandry [13,14,15]. Despite the GS success stories, studies on the application of genomic models in sorghum are limited compared to other cereal crops, such as maize, wheat, and rice [16]. For instance, a first genomic selection study in grain sorghum was reported by Hunt et al. [17] for prediction of test-cross performance in individual trials. Velazco et al. [12] investigated different genomic models including pedigree information for across-environment prediction of parental breeding values in productivity and adaptability traits [10].

Most of genomic selection algorithms implemented thus far were based on the analysis of single traits, while selection index has had limited use in actual plant breeding programs [18,19,20]. Nonetheless, available results indicate that selection index for improving a single trait would not outperform direct selection for the trait itself, whereas selecting simultaneously for more than one trait in selection index might outperform selecting for a single trait [20]. With the use of selection indices, individuals with very high merit in some traits are saved for breeding, even when they are slightly inferior in other traits [19,21], which can not only sustain productivity but can also safeguard genetic diversity. The selection index represents a joint analysis of multiple traits and can increase the accuracy of genetic evaluations in comparison with the single-trait analysis as it exploits the information from correlated traits [22].

The genomic selection index (GSI) is a linear combination of genomic estimated breeding values (GEBVs) used to predict the individual net genetic merit upon which individual candidates are selected from a nonphenotyped testing population as parents of the next selection cycle [19]. The efficiency of applying selection index in breeding depends on the strength of genetic and environmental correlations between the characters of interest. According to Thompson and Meyer [23], the benefit of selection index increases for lowly heritable traits, when analyzed together with strongly correlated traits of higher heritability. Another selection index advantage is represented by the possibility to reduce selection bias or culling bias introduced by contemporary or sequential selection on correlated traits, which are ignored by single-trait approaches [24]. The importance of selection index in genomic selection was demonstrated in empirical and simulation studies [3,25,26]. It was shown that genomic selection index models can efficiently be used to integrate information from correlated traits and from relatives. For this purpose, a breeder interested in response to selection for a single target trait, can incorporate other auxiliary traits in the index to provide additional information on the primary trait.

The efficiency of genomic selection index (GSI) models was shown in other cereal crops including maize, rice, and wheat [27,28,29]. In sorghum, GSI was implemented only in advanced breeding lines of grain sorghum [12] and in biomass-type genotypes using a pre-breeding population [3]. In the later work, the objective was to apply the GSI on auxiliary characters to indirectly predict the genomic estimated breeding value corresponding to the primary trait. In this work, we present, for the first time, a study we conducted on the potential of exploiting selection index for genomic selection in a panel of 380 biomass sorghum genotypes consisting of a mixture *Sorghum bicolor* landraces and lines and *S. bicolor* × *S. halepense* advanced inbred lines. Our objectives were to: (1) investigate if the use of a genomic selection index made up of aboveground dry biomass yield, dry mass fraction of the fresh mass material, and plant height can improve prediction accuracy relative to a single trait genomic selection index, and (2) investigate the efficiency of genomic selection indices in *S. bicolor* × *S. halepense* regrown from the rhizomes (overwintered testing set) using the populations grown from seeds as training set.

## 2. Materials and Methods

### 2.1. Phenotypic and Genotypic Data

Plant materials evaluated in this work belonged to a panel of 369 biomass sorghum genotypes of which 180 *Sorghum bicolor* landraces and lines and 189 *S. bicolor* × *S. halepense* advanced recombinant inbred lines beyond the F_7_ filial progeny. The two populations were evaluated at the same experimental site. Field trials covered four years (2014–2017 for *S. bicolor* and 2015–2018 for *S. bicolor* × *S. halepense*) for each population and were run side-by-side, except for in 2014 where only a *Sorghum bicolor* trial was planted. For the *S. bicolor* × *S. halepense* trials, the entire population was evaluated each year except in 2015 where only half the population was sown owing to scarce seed availability. Overall, there were four trials for *S. bicolor* population and nine trials for *S. bicolor* × *S. halepense* population. Of the nine trials of the later population, six were plants regrown from overwintered rhizomes, while three were trials grown from seeds. The list and the sizes (number of tested genotypes excluding checks) of the trials evaluated for each trait are presented in Table 1.

All the experiments were open-field trials and were established at CREA Research Center for Cereal and Industrial Crops, in the experimental station of Cà Rossa in Anzola (Bologna, Italy). The augmented randomized complete block design was used with six controls (checks) and six blocks [30] except US15 trials which had four checks and 4 blocks. Elementary plots were single 5 m long rows distant 0.75 m, and were thinned to homogeneously distributed 50 plants per plot. We evaluated open field morpho-physiological data on aboveground dry biomass yields (DMY, t ha^−1^), plant height (PH, cm), and dry mass fraction of the fresh material (DMC, %), as suggested by IBPGR [31]. Plant height was measured one week before harvest as the mean height of the elementary plot using a 5 m telescopic rod (Stanley 5 m grade rod aluminium) placed vertically on the ground in the middle of the row. Aboveground dry biomass yield and the dry mass fraction of the fresh material were measured as follows. The entire plot was machine chopped and fresh weight plot yield scored. Immediately after a plot was weighed, a sample was taken from each plot then weighed before and after oven drying at 80 °C to constant weight to determine moisture content. Dry mass fraction of the fresh material (DMC%) = (sample dry weight/sample wet weight) × 100. Aboveground dry biomass yield in metric tons per hectare (DMY t ha^−1^) = ((total plot wet weight (kg) × (sample dry weight/sample wet weight))/ plot area (m^2^)) × 10.

### 2.2. Phenotypic Data Analysis

Data from single trials were analyzed in two steps. In the first step, the adjusted means were calculated as suggested by Federer [30] to account for the variability of soil properties. In the second step, adjusted means from each trial were jointly analyzed to estimate genotype means across environments. The model fitted was as follows: $y_{ik}\;=\;\mu +G_{i}+E_{k}+GE_{ik}+\varepsilon_{ik}$, where $y_{ik}$ is the best linear unbiased estimation (BLUE) of *i*-th genotype in the *k*-th environment, which was fitted by a random genotype effect ($G_{i}$), a fixed environmental effect ($E_{k}$), and the genotype × environment interaction ($GE_{ik}$). Given that genotype effects were considered random, the GE interaction involving $G_{i}$ was random. All random effects were assumed independent homoscedastic and normally distributed with zero mean. The best linear unbiased estimates were used in the subsequent processes of fitting the genomic selection models.

### 2.3. Molecular Data

DNA extraction and whole-genome genotyping procedures were amply described in Habyarimana and Lopez-Cruz [10]. The molecular information used in this work consisted of genotyping-by-sequencing single nucleotide polymorphisms (GBS SNPs) produced by BGI Hong Kong Company Limited. To prepare the library, the ApeKI, a methylation-sensitive restriction enzyme, was used, and GBS was carried out on an Illumina HiSeq X Ten platform. For variants discovery, the sequencing reads were aligned to the sorghum reference genome (*Sorghum_bicolor* NCBIv3). The SNP datasets were filtered using VCF tools to extract marker data responding to high quality standards such as biallelic SNPs only, minor allele frequency (MAF) ≥ 0.05, site quality or the Phred-scaled probability that reference/alternative alleles polymorphism exists at a given site given the sequencing data Q ≥ 40 (i.e., base call accuracy ≥ 99.99%), and missing genotypes (NA) ≤ 20%. The final size of the high quality-controlled marker dataset matrix was 61,976 SNPs which were used in downstream steps in this work for genomic prediction and selection analytics.

### 2.4. Construction of Genomic Selection Indices

In matrix notation, an optimum phenotypic selection index (PSI) [32] takes the following form [20] $I_{i}\;=\;\sum_{j\;=\;1}^{p}\beta_{j}x_{ij}\;=\;\beta^{\prime }x_{i}$ where $\beta^{\prime }\;=\;\left[\beta_{1}\beta_{2}\ldots .\beta_{p}\right]$ is a vector of coefficients, p is the number of traits on $I_{i}$, and $x_{i}\;=\;\left[x_{i1},\;\ldots ,\;x_{ip}\right]$ is a vector of p measured phenotypic values which are centered with respect to their respective means. The linear genomic selection index for individual i is represented by the aggregate genotypes H and was defined as $H_{i}\;=\;\sum_{j\;=\;1}^{t}\alpha_{j}g_{yij}\;=\;\beta^{\prime }g_{yi}$ where ${\overset{´}{g}}_{yi}\;=\;\left[g_{yi1}g_{yi2}\ldots .g_{yit}\right]$ is a vector of the genotypic values of t selection targets $y_{i}$ and $\alpha^{\prime }\;=\;\left[\alpha_{1}\alpha_{2}\ldots .,\alpha_{t}\right]$ a vector of known and fixed economic weights [19]. Under the breeding perspective, economic values are used to reflect the relative importance of the traits of interest. The economic value is the increase in profit achieved by improving a particular trait by one unit [33,34]. In case of several traits, the total economic value is a linear combination of the breeding values of the traits weighted by their respective economic values as in the above equation [19,32], and this is called the net genetic merit (or aggregate genotype, selection target) of one individual.

To be used in the optimum indices, the $\beta_{j}$ are derived such that $I_{i}$ is maximally correlated with $H_{i}$, the solution of which is found to be the following matrix equation [20,35] $\hat{\beta }\;=\;P_{x}^{-1}G_{x,y}\alpha$. The matrices $G_{x,y}$ and $P_{x}$ are, respectively, the genotypic covariance between the measured phenotypes and the selection targets, and the phenotypic variance-covariance among the measured phenotypes. On the other hand, $\hat{\beta }$ is the best linear unbiased predictor (BLUP) of $\beta_{j}$, while α is as described above [32,36,37]. From the above equations, the following statistics were derived as suggested in [18,19]: (1) heritability of the index $h_{I}^{2}\;=\;\beta^{\prime }G_{x}\beta /\beta^{\prime }P_{x}\beta$, where $G_{x}$ is the genotypic variance-covariance matrix among the measured phenotypes, (2) genetic correlation between the index and the selection target $gencor\;=\;cor\left(g_{I},g_{H}\right)\;=\beta^{\prime }G_{x,y}\alpha /\sqrt{\beta^{\prime }G_{y}\alpha }\sqrt{\beta^{\prime }G_{x}\beta }$, where $G_{y}$ is the genotypic variance-covariance matrix among the selection targets, and (3) accuracy of selection defined as the correlation between the index and the genotypic value of the selection target i.e., $acc\;=\;cor\left(I,g_{H}\right)=\;cor\left(g_{I},g_{H}\right)h_{I}$. The accuracy of selection was used to evaluate the performance of the genomic prediction model performance.

### 2.5. Genomic Selection Models

In the genomic selection index modeling, phenotypic and marker data are scored in the training population and fitted into appropriate algorithm to produce individuals’ whole-genome marker effects. The marker effects are used in subsequent cycles of selection to compute the genomic estimated breeding values (GEBVs) that are used as predictors of breeding values in a testing unphenotyped population. The genomic estimated breeding values are obtained as a product of the estimated marker effects in the training population and the coded marker values obtained in the testing population. To apply genomic selection index, GEBVs are obtained in the selection candidates and then used to predict and rank the net genetic merit of the candidates for selection.

In this work, the genomic selection analyses were implemented in the multiple-trait model (MTM) software [38] that uses a Bayesian approach [39]. The routines built in the MTM package allow the calculation of the phenotypic and genotypic variance-covariance matrices. The performance of the genomic selection models was assessed using Monte Carlo (repeated hold-out) cross-validation approach [40,41] applying 70% and 30%, respectively, as training and validation (test) sets. In a standard hold-out cross-validation, the test set represents new, unseen data to the model. To obtain a more robust performance estimate that was less dependent on how the data was split into training and validation sets, the holdout method was repeated 100 times using different random seeds. The hundred repetitions were then used to calculate the average prediction performance. In comparison to the standard holdout validation method, the repeated hold-out procedure implemented in this work provides a better estimate of the model prediction ability when a random test set is used [41]. The repeated hold-out procedure provides also the information about the stability of the model (produced by a learning algorithm) across training set splits. The parameters of the models were estimated in the training set before the models were validated in the testing set. The performance of the models was measured using the accuracy of selection and the genetic correlation between the index and the selection target as described previously [18,19].

**Figure 1:**
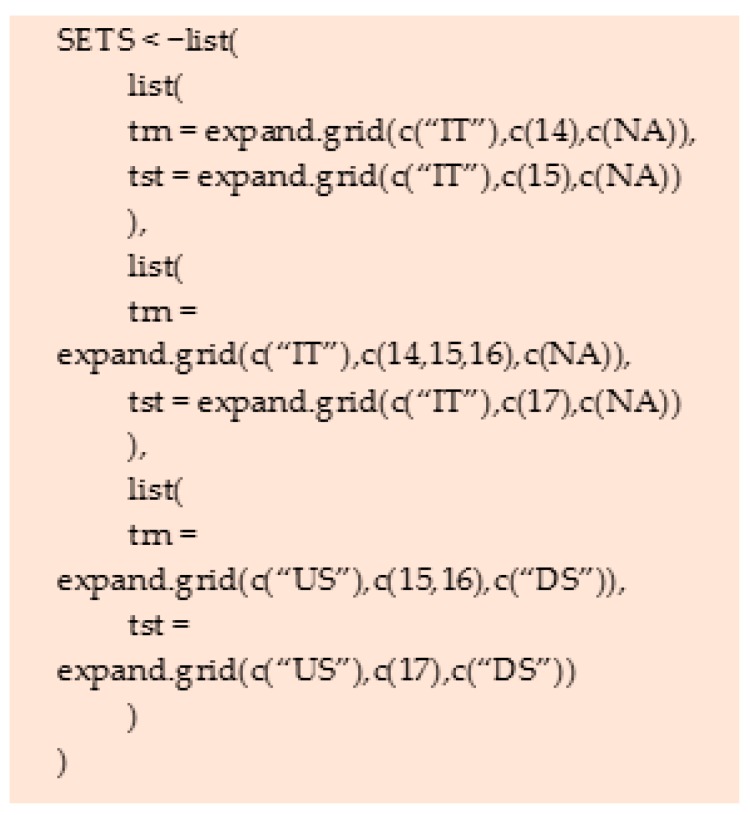
Example of a code snippet instructing the creation of training and testing sets. The list called ‘SETS’ contains three different training-testing (TRN-TST) sets. The IT14, IT15, IT16, and IT17 are four S. bicolor trials containing same genotypes evaluated in 2014, 2015, 2016, and 2017, respectively. The first set will train the model using as TRN set IT14 to predict IT15. In the second scenario, the model will be trained in IT14 + IT15 + IT16 to predict IT17, while in the third scenario, the model will be trained in US15DS + US16DS to predict US17DS.

The selection index algorithms were implemented for different targets of prediction considering $H_{i}\;=\;g_{yij}$ for each single trait in the target set, and then $H_{i}\;=\;\sum_{j\;=\;1}^{t}\alpha_{j}g_{yij}$ for multi-trait genomic selection index, with $\alpha^{\prime }=\;\left[1,\;\ldots ,\;1\right]$ representing the economic weights of the t traits for which we expressed equal preference [32,35]. In the box below (Figure 1) is the example of a code snippet used in this work to instruct the creation of a training and testing sets in R:

The models were implemented using R software, version 3.5.3 (R Core Team, Vienna, Austria) [42] and the package MTM [1,38] by applying default rules for selecting hyperparameters. The Gibbs sampler was used and our analyses were based on 30,000 samples from the posterior distribution obtained after the first 5000 iterations were discarded as burn-in [1]. The visualization algorithms and statistical inferences used to present the genomic selection models’ output were implemented using routines called from the R software. The magnitude and direction of the Pearson correlation coefficients were interpreted according to Gomez and Gomez [43] as follows: 0–0.1, 0.1–0.5, 0.5–0.8, and 0.8–1, 1, respectively, zero, low, medium, high, and perfect.

## 3. Results

### 3.1. Comparison of Traits, Genetic Metrics, and Genomic Selection Approaches

**Figure 2:**
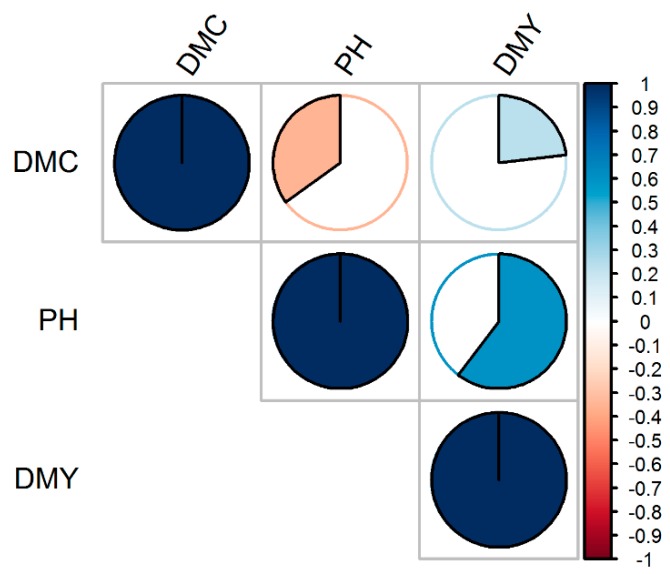
Pearson correlation coefficients among the evaluated traits. DMC, DMY, and PH, respectively, denote dry mass content, dry biomass yield, and plant height. The filled-in areas of the circles show the absolute value of corresponding correlation coefficients. The scale on the right hand side is colored from red (negative correlation) to blue (positive correlation); with the intensity of color scaled 0%–100% in proportion to the magnitude of the correlation. Refer to text for the description of the traits.

**Figure 3:**
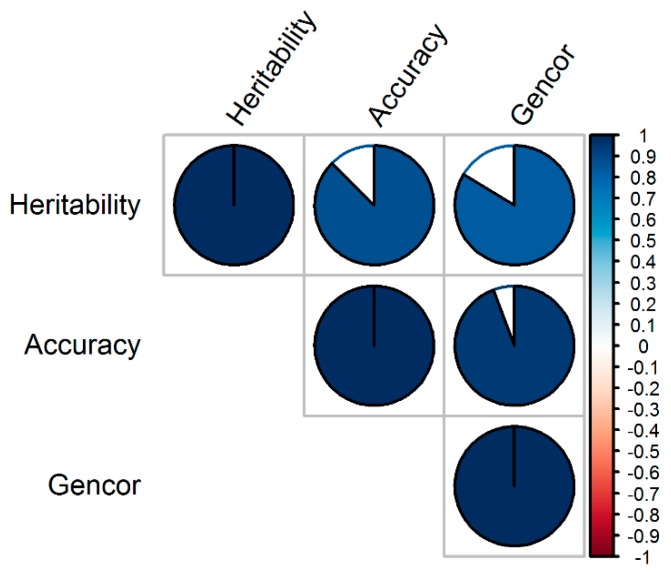
Pearson correlation coefficients among the genetic metrics. Gencor genetic correlation between the phenotypic and the genomic selection indices. The filled-in areas of the circles show the absolute value of corresponding correlation coefficients. The scale on the right-hand side is colored from red (negative correlation) to blue (positive correlation); with the intensity of color scaled 0%–100% in proportion to the magnitude of the correlation. Refer to text for the description of the genetic metrics.

**Figure 4:**
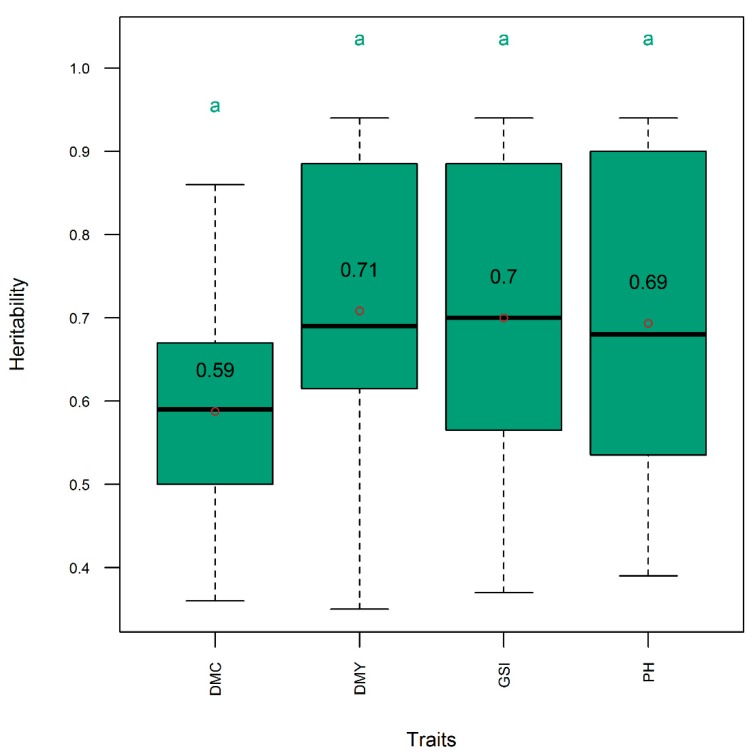
Distribution (boxplot) of narrow-sense heritability for single trait and three-trait selection indices in the entire panel. DMC, DMY, GSI, and PH, respectively, denote selection indices relative to dry mass fraction of fresh material, aboveground dry biomass yield, three-traits (DMC, DMY, and PH), and plant height. Means are indicated by open dots and are included within the boxplot. Means with same letter are not significantly different at the 5% level using the Tukey′s HSD (honestly significant difference) test. Refer to text for the description of the GS models.

**Figure 5:**
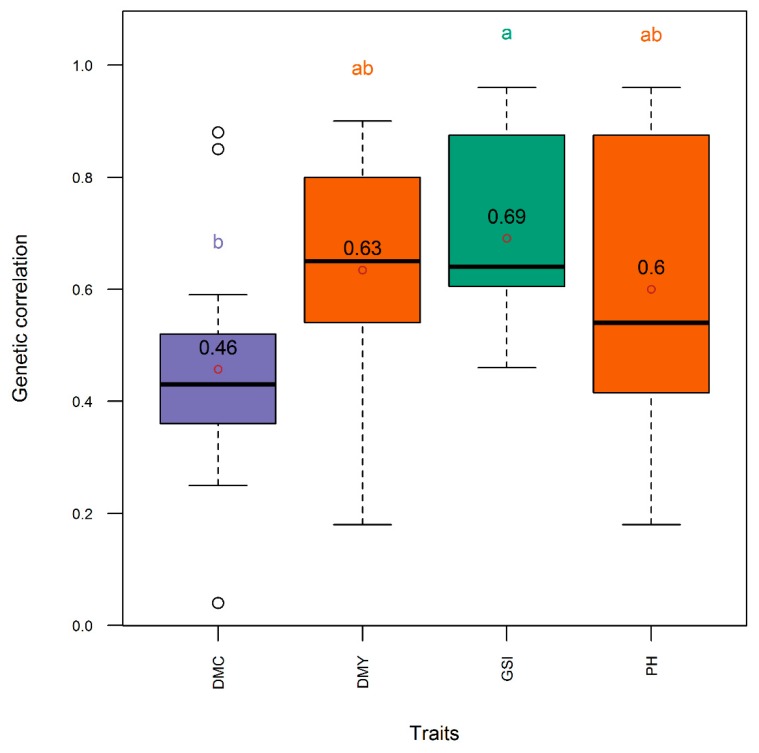
Distribution (boxplot) of genetic correlation between the phenotypic indices and the net genetic merit in the entire panel. DMC, DMY, GSI, and PH, respectively, denote selection indices relative to dry mass fraction of fresh material, aboveground dry biomass yield, three-trait (DMC, DMY, PH), and plant height. Means are indicated by open dots and are included within the boxplot. Means with same letter are not significantly different at the 5% level using the Tukey’s HSD (honestly significant difference) test. Refer to text for the description of the GS models.

**Figure 6:**
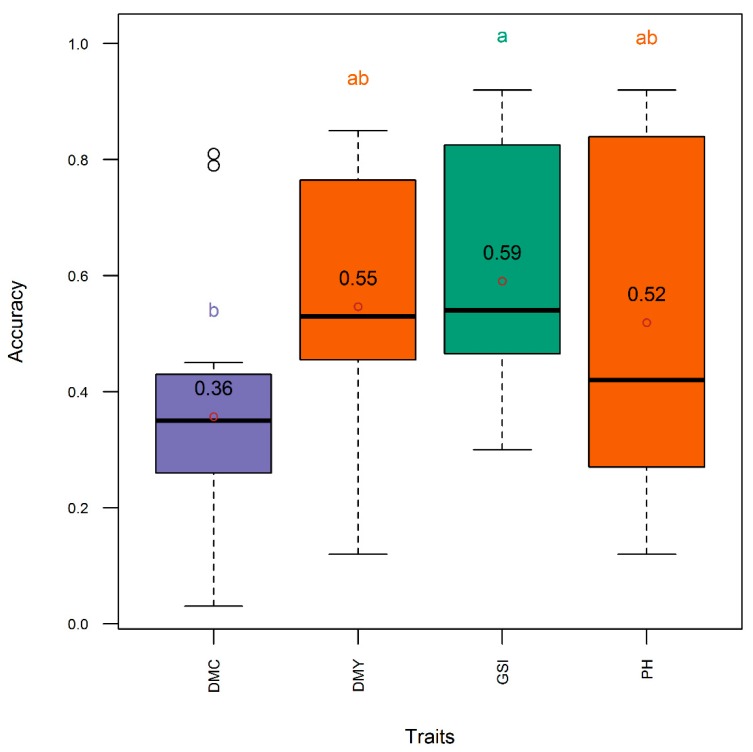
Distribution (boxplot) of genomic selection index accuracy using single traits and all three traits of interest simultaneously in the entire panel. DMC, DMY, GSI, and PH, respectively, denote selection indices relative to dry mass fraction of fresh material, aboveground dry biomass yield, all the three traits simultaneously, and plant height. Means are indicated by open dots and are included within the boxplot. Means with same letter are not significantly different at the 5% level using the Tukey′s HSD (honestly significant difference) test. Refer to text for the description of the GS models.

The Pearson correlation was low and negative (*r* = −0.35) between the dry mass fraction of the fresh material and the plant height, low and positive (*r* = 0.23) between dry mass fraction of the fresh material and the aboveground dry biomass yield, and medium and positive (*r* = 0.60) between plant height and the aboveground dry biomass yield (Figure 2). On the other hand, the Pearson correlation was higher (*r* = 0.94) between accuracy and genetic correlation, followed by the correlation between accuracy and heritability (*r* = 0.87), and between heritability and genetic correlation (*r* = 0.84) (Figure 3). The heritability of all single traits and the genomic selection index came from same distribution with statistically comparable means (*h*^2^ = 0.59–0.71) (Figure 4). Genetic correlation was higher (gencor = 0.6–0.63) and comparable in genomic selection index, aboveground dry biomass yield and plant height, while it was lower (gencor = 0.46) for the dry mass fraction of the fresh weight (Figure 5). The accuracy showed the same pattern as the genetic correlation. The accuracy was higher (*acc* = 0.52–0.59) and comparable in genomic selection index, aboveground dry biomass yield and plant height, while it was lower (*acc* = 0.36) for the dry mass fraction of the fresh weight (Figure 6).

**Figure 7:**
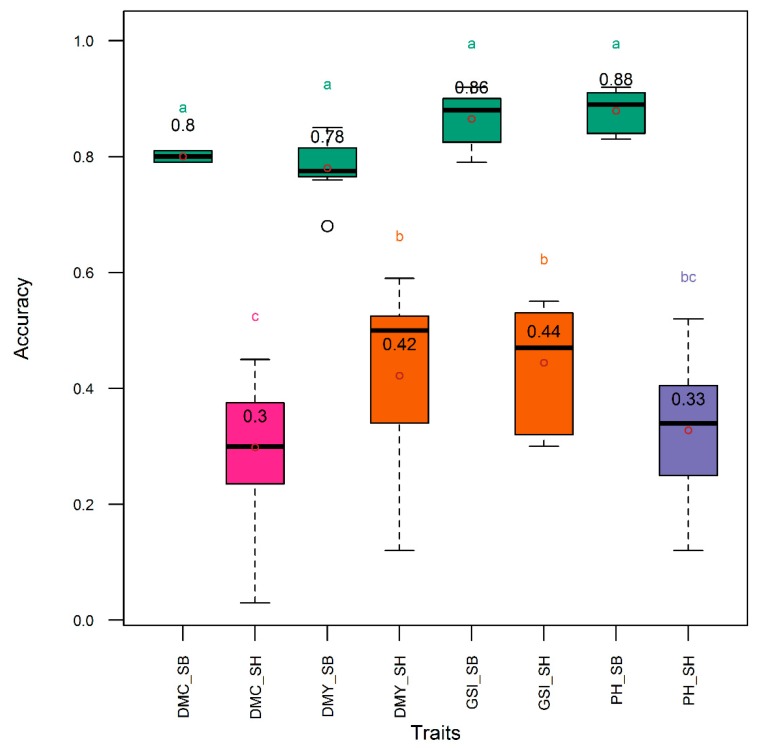
Distribution (boxplot) of genomic selection accuracy using single traits and all traits simultaneously in *Sorghum bicolor* and *S. bicolor* × *S. halepense* lines. DMC, DMY, GSI, and PH, respectively, denote selection indices relative to dry mass fraction of fresh mass material, aboveground dry biomass yield, all the three traits simultaneously, and plant height. Traits suffixed with “_SB” and “_SH”, respectively, were collected from *Sorghum bicolor* and *S. bicolor* × *S. halepense* lines. Means are indicated by open dots and are included within the boxplot. Means with same letter are not significantly different at the 5% level using the Tukey’s HSD (honestly significant difference) test. Refer to text for the description of the GS models.

The heritability and the genetic correlation were higher in *Sorghum bicolor* than in *S. bicolor* × *S. halepense*. Genetic correlation in *Sorghum bicolor* vs. *S. bicolor* × *S. halepense* was 0.86 vs. 0.40, 0.82 vs. 0.54, 0.92 vs. 0.43, and 0.91 vs. 0.57, respectively for the dry mass fraction of the fresh material, aboveground biomass yield, plant height, and the three-trait genomic selection index. Heritability in *Sorghum bicolor* vs. *S. bicolor* × *S. halepense* was 0.86 vs. 0.55, 0.90 vs. 0.60, 0.92 vs. 0.57, and 0.91 vs. 0.59, respectively for the dry mass fraction of the fresh material, aboveground biomass yield, plant height, and the three-trait genomic selection index. Accuracy in the *Sorghum bicolor* subpopulation was higher than in the *S. bicolor* × *S. halepense* subpopulation for all traits and the genomic selection index (Figure 7). In *S. bicolor*, accuracy was comparable (*acc* = 0.78–0.88) among single traits and the genomic selection index, while in in *S. bicolor* × *S. halepense*, the pattern followed that of the entire diversity panel with higher and comparable accuracy (*acc* = 0.33–0.44) in genomic selection index, aboveground dry biomass yield and plant height, while the accuracy was lower (*acc* = 0.30) for the dry mass fraction of the fresh weight (Figure 7).

### 3.2. Predicting Regrowth Performance in Perennial Sorghum Bicolor × Sorghum Halepense

**Figure 8:**
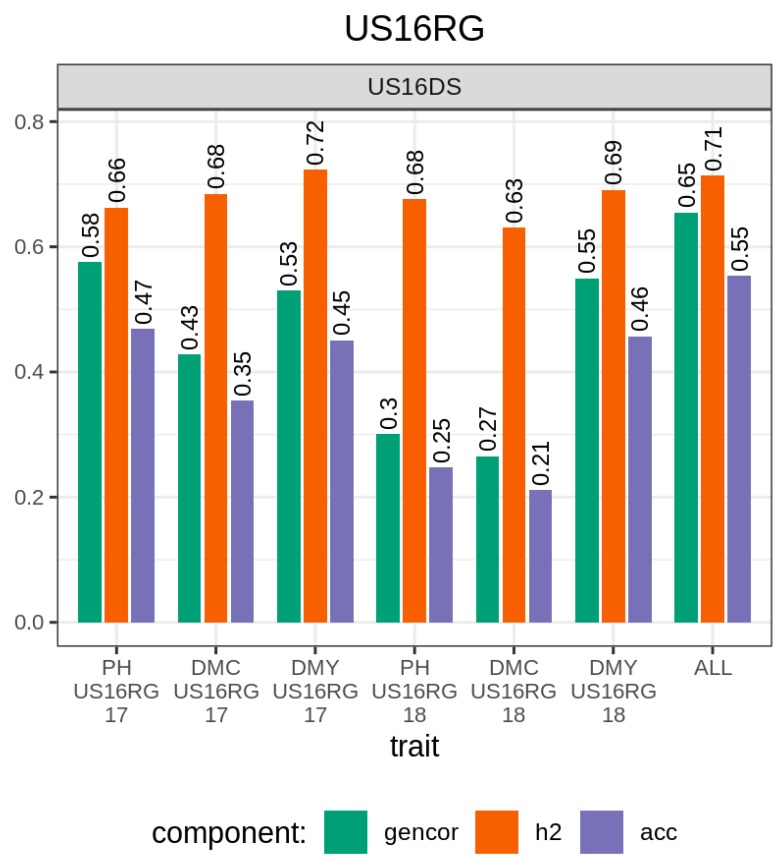
Accuracy of genomic prediction of the performance of *S. bicolor* × *S. halepense* regrown from rhizomes (testing set) using trials grown from seeds as training set. US16DS *S. bicolor* × *S. halepense* sown and evaluated in 2016; US16RG17 *S. bicolor* × *S. halepense* sown in 2016 and regrown and evaluated in 2017; US16RG18 *S. bicolor* × *S. halepense* sown in 2016 and regrown and evaluated in 2018. DMC, DMY, ALL, and PH, respectively, denote selection indices relative to dry mass fraction of the fresh material, aboveground dry biomass yield, all the three traits simultaneously, and plant height. Numbers on the top of the bars are mean accuracies. Refer to text for the description of the GS models.

The information from the *Sorghum bicolor* × *Sorghum halepense* trial sown in 2016 was used to predict the performance of the overwintered (regrowth) populations in 2017 and 2018 (Figure 8). For plant height, genetic correlation and accuracy were 0.58 and 0.47, respectively, in 2017 and decreased by 48% and 47%, respectively, in 2018. For the dry mass fraction of the fresh mass material, genetic correlation and accuracy were 0.43 and 0.35, respectively, in 2017 and decreased by 37% and 40%, respectively, in 2018. For the aboveground dry biomass yield, the genetic correlation and accuracy remained stable from 2017 to 2018 with respective ranges of 0.53–0.55 and 0.45–0.46. The heritability of the above three traits remained stable from 2017 to 2018 decreasing or increasing by one to five hundredths. The genetic correlation and accuracy obtained with the genomic selection index were higher than the best values obtained with a single trait. On the other hand, the heritability obtained with the genomic selection index was comparable to that obtained with the aboveground dry biomass and higher than the heritability realized in other traits.

## 4. Discussion

A diversity panel made up of a mixture of *Sorghum bicolor* lines and landraces and *Sorghum bicolor* × *Sorghum halepense* advanced recombinant inbred lines was used in this work in order to set up the groundwork upon which to build future germplasm improvement and cultivar development programs. A similar panel was used previously in a genome-wide linkage disequilibrium investigation in sorghum, and in genomic prediction and selection for antioxidant production in sorghum [10,44]. In these previous studies, mixing *Sorghum bicolor* and *Sorghum bicolor* × *Sorghum halepense* genotypes was motivated mainly by the observed weak structure of the resulting diversity panel. In addition, in these investigations and in the present work, *Sorghum bicolor* relevant information was used as the molecular marker information used was derived by aligning the sequencing reads to the sorghum reference genome (Sorghum_bicolor NCBIv3) to enable variants discovery. It was also shown that the use of *Sorghum bicolor* × *Sorghum halepense* recombinant inbred lines in the diversity panel brought novel useful polymorphism [44].

The correlation observed among the evaluated traits was not in full agreement with Habyarimana et al. [1] except for the relationship between plant height and the aboveground dry biomass yield. The differences between the two works can be attributed to different types of populations evaluated. In this work a panel of *Sorghum bicolor* and *S. bicolor* × *S. halepense* was evaluated, while the correlation reported in Habyarimana et al. [1] referred only to *S. bicolor* × *S. halepense*. The high pairwise correlation between plant height and the aboveground dry biomass yield implied that the proportion of variance shared by these traits was mostly explained by genetic causes. A perfect correlation between plant characteristics implies that genetic effects on the traits of interest are identical, which can indicate the existence of either linkage disequilibrium, pleiotropy or causal overlap, or ascertainment bias deriving from biased sampling [10].

The lower correlation coefficients observed in this work between the dry mass fraction of the fresh material and the plant height, on the one hand, and the aboveground dry biomass yield on the other hand implies that dry mass fraction of the fresh material can be improved independently of plant height and aboveground dry biomass yield. This can have important implications in terms of sustainability because high-yielding genotypes can be bred that contain less moisture in biomass at harvest, which means less energy would be spent on biomass conversion and transportation from the field to the bioreactor.

When faced with the necessity to simultaneously improve more than one trait, a breeder can use three approaches: tandem selection, independent culling levels, and index selection [45]. In tandem selection, only one character is selected in each cycle; in independent culling levels, all genotypes with a phenotypic value below the culling threshold for at least one characteristic are discarded; the selection index aims at improving several traits simultaneously in such a way as to make the biggest possible improvement in overall genetic merit [35]. In this work, we implemented the Optimum selection Index of Smith [32] the performance of which was demonstrated in previous studies [35,37]. In our optimum index selection, both desirable and undesirable (e.g., plant height vs. dry mass fraction of the fresh material) correlations were observed between traits (Figure 2) but, as Bradshaw [35] put it, these were accommodated by the index accounting for the simultaneous improvement of the traits on the index. In the process of computing the optimum index selection, equal weights in terms of phenotypic standard deviations ($1/\sigma_{P}$) were used as suggested by Bradshaw [35] and supported by Saeidnia et al. [46]. The later authors used optimum index and compared several economic weights including unit, phenotypic correlation, genotypic correlation, heritability, direct effects in path analysis and first factor loading in factor analysis. They found out that using unit coefficient in the optimum selection index allowed the highest genetic advance for all traits making up the index. In the same work the selection index with equal weights showed high correlation with the net genetic merit.

The accuracy was more associated with genetic correlation than heritability because heritability was generally high and did not show high variability across trials. This relationship among heritability, genetic correlation and accuracy of selection was consistently observed both in Pearson correlation analysis (Figure 3) and in post hoc analytics through mean separation (Figure 2, Figure 3 and Figure 4). From the high heritability values of the index selection it can be inferred that the indices described in this work can be effectively used in breeding programs without significant environmental noise. The genetic correlation and accuracy were statistically comparable between the three-trait index selection, aboveground dry biomass yield and plant height, but these metrics were significantly lower for the dry mass fraction of the fresh material (Figure 3 and Figure 4). It can therefore be inferred that the use of the three traits in the index selection can simultaneously improve the accuracy for selecting aboveground dry biomass yield, plant height, and particularly, the dry mass fraction of fresh material. Indeed, this is the inherent characteristics of a linear selection index as it is expected to allow extra merit in one trait to offset defects that existed in another. As Hazel and Lush [19,21] showed, by the use of a linear selection index, individuals with very high merit in one trait are saved for breeding, even when they are inferior in other traits.

The higher accuracy of selection observed in *Sorghum bicolor* relative to *S. bicolor* × *S. halepense* can be explained by the lower genetic variability in the *S. bicolor* × *S. halepense* materials as confirmed by their observed lower heritability of the index and lower genetic correlation between the index and the net genetic merit. The low genetic variability in *S. bicolor* × *S. halepense* lines might have resulted from the low number of parents used during early hybridizations [47] that led to a relatively narrow genetic base in the current progeny. On the other hand, higher genetic variability in *S. bicolor* was expected as these genotypes were derived from African and Asian landraces, and are expected to harbor a high level of genetic diversity for breeding purposes inasmuch as Africa and Asia represent, respectively, the primary and secondary sorghum centers of diversity [2].

The results from the regrowth trials were encouraging. Heritability was consistently higher across years for all selection indices, implying that effective selection can be carried out even several overwintering generations after the original seed sown trials. Among single trait genomic selection indices, the aboveground dry biomass yield showed better accuracy relative to other traits, and maintained the good accuracy across years. The accuracy for the dry mass fraction of the fresh material and the accuracy for plant height decreased over years. For these traits, the accuracy in regrowth trials can probably be improved by either re-training the models including the information from the immediately precedent generation or integrating the single traits of interest in a multi-trait index selection. The observed higher accuracy for the three-trait genomic selection holds therefore good promise for improving aboveground dry biomass yields and its auxiliary traits like plant height and the dry mass fraction of the fresh material in *S. bicolor* × *S. halepense*.

## 5. Conclusions

In this work, extensive experimental breeding data were used to demonstrate for the first time that the optimum index selection can be implemented in genomic selection predictive analytics for index selection including aboveground dry biomass yield, plant height, and dry mass fraction of the fresh material in biomass sorghum crop. Furthermore, this work shed light for the first time on the promising potential of using the information from the trial grown from seed to predict the performance of the populations regrown from the rhizomes even two winter seasons after the original trial was sown. For these particular populations established from regrowths, using multi-trait index selection was the recommended option to improve traits such as plant height and the dry mass fraction of the fresh material that were weakly predicted when the selection target was regrown from the rhizomes. Since the plant characteristics evaluated herein are routinely measured in cereal and other plant species of agricultural interest, it can be inferred that our findings can be harnessed in other major crops as well.

## Figures and Tables

**Table 1:** Trials and respective sizes of populations and the traits evaluated.

Trials	PH	DMC	DMY ^1^
IT14	174	123	123
IT15	179	179	179
IT16	180	NA	180
IT17	168	168	168
US15_DS	90	90	90
US15_RG16	89	89	89
US15_RG17	85	85	85
US15_RG18	85	85	85
US16_DS	189	189	189
US16_RG17	189	189	189
US16_RG18	189	189	189
US17_DS	189	189	189
US17_RG18	189	189	189

^1^ IT and US, respectively, denote *S. bicolor* and *S. bicolor* × *S. halepense* trials. DS, RG, PH, DMC, DMY, respectively, denote trials grown from seeds (direct sowing trials), trials regrown from overwintered rhizomes (regrowth trials), plant height, dry mass fraction of the fresh material, and aboveground dry biomass yield. Number following IT and US are the years of direct sowing trials, while the numbers following RG are the years of the regrowth trials. “NA” indicates that the data was not scored.
